# Incidence, characteristics, and outcomes of delirium in patients with noninvasive ventilation: a prospective observational study

**DOI:** 10.1186/s12890-021-01517-3

**Published:** 2021-05-11

**Authors:** Rui Zhang, Linfu Bai, Xiaoli Han, Shicong Huang, Lintong Zhou, Jun Duan

**Affiliations:** Department of Respiratory and Critical Care Medicine, The First Affiliated Hospital of Chongqing Medical University, Youyi Road 1, Yuzhong District, Chongqing, 400016 China

**Keywords:** Delirium, Noninvasive ventilation, Risk factor

## Abstract

**Background:**

Factors that may increase the risk for delirium and the firm knowledge around mechanism for delirium in noninvasive ventilation (NIV) patients is lacking. We investigated the incidence, characteristics, and outcomes of delirium in NIV patients.

**Methods:**

A prospective observational study was performed in an intensive care unit (ICU) of a teaching hospital. Patients in whom NIV was used as a first-line intervention were enrolled. During NIV intervention, delirium was screened using the Confusion Assessment Method for the ICU each day. The association between delirium and poor outcomes (e.g., NIV failure, ICU and hospital mortality) was investigated using forward stepwise multivariate logistic regression analyses.

**Results:**

We enrolled 1083 patients. Of these, 196 patients (18.1%) experienced delirium during NIV intervention. Patients with delirium had higher NIV failure rates (37.8% vs. 21.0%, *p* < 0.01), higher ICU mortality (33.2% vs. 14.3%, *p* < 0.01), and higher hospital mortality (37.2% vs. 17.0%, *p* < 0.01) than subjects without delirium. They also had a longer duration of NIV (median 6.3 vs. 3.7 days, *p* < 0.01), and stayed longer in the ICU (median 9.0 vs. 6.0 days, *p* < 0.01) and the hospital (median 14.5 vs. 11.0 days, *p* < 0.01). These results were confirmed in COPD and non-COPD cohorts. According to subtype, compared to hyperactive delirium patients, hypoactive and mixed delirium patients spent more days and many more days on NIV (median 3.4 vs. 6.5 vs. 10.1 days, *p* < 0.01). Similar outcomes were found for length of stay in the ICU and hospital. However, NIV failure, ICU mortality, and hospital mortality did not differ among the three subtypes.

**Conclusions:**

Delirium is associated with increases in poor outcomes (NIV failure, ICU mortality, and hospital mortality) and the use of medical resources (duration of NIV, and lengths of stay in the ICU and hospital). Regarding subtype, hypoactive and mixed delirium are associated with higher, and much higher, consumption of medical resources, respectively, compared to hyperactive delirium.

**Supplementary Information:**

The online version contains supplementary material available at 10.1186/s12890-021-01517-3.

## Background

Delirium is characterized by acute onset of cerebral dysfunction with a change or fluctuation in baseline mental status, inattention, and either disorganized thinking or altered consciousness [1]. Delirium is divided into hyperactive, hypoactive, and mixed subtypes [2]. It frequently occurs in critically ill patients due to effects of their acute illness as well as pain, stress, anxiety, sleep deprivation, sedation, analgesia, and antimicrobial exposure [3–7]. And its pathophysiology has not yet been clearly established.

A systematic review and meta-analysis reported that the pooled incidence of delirium is 31.8% in critically ill patients [8]; it is reportedly much higher (reaching 80%) in mechanically ventilated patients [9]. Delirium is associated with prolonged length of stay (LOS) in the ICU and the hospital, increased hospital mortality, and elevated hospital costs [8, 10–12]. Many strategies to prevent delirium have been investigated [13–15]. However, such studies have generally focused on mechanically ventilated or surgical patients. Knowledge of delirium in patients who have received noninvasive ventilation (NIV) is lacking.

In this study, we investigated the incidence, clinical characteristics, and outcomes of delirium in NIV patients, as well as the distribution of its different subtypes and the association between delirium and NIV failure.

## Methods

### Patients

This was a prospective observational study performed in an ICU of a teaching hospital from 2016 to 2018. The study protocol was approved by the hospital’s institutional review board. Due to the observational nature of this study, informed consent was waived. Patients who were admitted to the ICU for NIV as a first-line intervention were enrolled, and patients younger than 18 years old were excluded. Patients who were treated with NIV after high-flow nasal cannula or invasive mechanical ventilation were also excluded.

### NIV management

NIV (BiPAP Vision or V60; Philips Respironics, Carlsbad, CA) was initiated by the attending physicians in relation to previously described indications [16, 17]. In patients with chronic obstructive pulmonary disease (COPD), the indications for NIV were respiratory rate more than 25 breaths/min, PaCO_2_ more than 45 mmHg, pH less than 7.35, PaO_2_/FiO_2_ less than 200 mmHg, and vigorous activity of the accessory respiratory muscles. In patients with hypoxemic respiratory failure, the indications for NIV were clinical presentation of respiratory distress at rest (such as active contraction of the accessory inspiratory muscles or paradoxical abdominal motion) and PaO_2_/FiO_2_ less than 300 mmHg.

A face mask (ZS-MZ-A Face Mask, Shanghai Zhongshan Medical Technology Co., Shanghai, China) was the first choice to connect the patient to the ventilator. The size of the mask was chosen to fit the patients’ face type. The S/T mode was used in COPD patients or other patients with labored breathing. The initial inspiratory positive airway pressure was 8–10 cmH_2_O, which was gradually increased to reach a tidal volume of 6–8 mL/kg or the maximal tolerated level. The initial expiratory positive airway pressure was 4 cmH_2_O. This was gradually increased to counterbalance the intrinsic positive end expiratory pressure in COPD patients and titrated according to the flow curve to ensure that expiratory flow reached zero prior to inspiration or diminished ineffective efforts. In patients with hypoxemic respiratory failure, it was increased to maintain alveoli patency and to elevate the end expiratory lung volume. In patients with heart failure, continuous positive airway pressure was used if the PaCO_2_ less than 45 mmHg and S/T was used if PaCO_2_ more than 45 mmHg. In all patients, the ventilator parameters were also adjusted according to the actual PaCO_2_ and PaO_2_. The fractional concentration of oxygen was set to reach a peripheral oxygen saturation of > 92%.

At the beginning of the treatment, continuous use of NIV was encouraged. Once the patient recovered from respiratory failure, liberation from NIV was considered, following our hospital protocols [18]. However, intubation was indicated if respiratory failure progressively deteriorated. The criteria for intubation were as follows: respiratory or cardiac arrest, failure to maintain PaO_2_/FiO_2_ more than 100, development of a condition necessitating intubation to protect the airway (coma or seizure disorder) or to manage copious tracheal secretions, inability to correct dyspnea, lack of improvement of signs of respiratory muscle fatigue, and hemodynamic instability without response to fluids and vasoactive agents [16, 17]. NIV failure was defined as requirement for intubation.

### Assessment of delirium

Delirium was screened using the Confusion Assessment Method for the ICU (CAM-ICU) every morning from NIV initiation to termination [9]. The assessment was performed by trained researchers. Delirium was assessed based on the following four features: (1) fluctuation in mental status, (2) inattention, (3) disorganized thinking, and (4) altered consciousness. Delirium was diagnosed in the presence of features 1 and 2 and either feature 3 or 4. The level of consciousness was assessed using the Richmond Agitation-Sedation Scale (RASS) [19]. RASS is a 10-point scale ranging from unarousable (–5 points) through calm (0 points) to combative (4 points). The three subtypes of delirium were defined as follows [20]. Hyperactive delirium was defined as present in patients with all positive daily RASS scores (range, 1–4 points) associated with every positive CAM-ICU assessment. Hypoactive delirium was defined as present in patients with all neutral or negative daily RASS scores (range, 0 to –3 points) associated with every positive CAM-ICU assessment. Mixed delirium was defined as present in patients with daily RASS scores that included both positive values (range, 1–4 points) and neutral or negative values (range, 0 to –3 points) associated with every positive CAM-ICU assessment.

### Outcomes

The primary outcome was the rate of NIV failure. The second outcome included ICU mortality, hospital mortality, duration of NIV, length of stay in the ICU, length of stay in the hospital, and delirium days. The data was collected before hospital discharge. If patient died during hospitalization, the data was collected at the day of death.

### Statistical analyses

Statistical software (SPSS 17.0; IBM Corp., Armonk, NY) was used to analyze the data. Continuous variables are presented as means with standard deviations or medians and interquartile ranges (25–75%) when appropriate. The normality of data distribution was analyzed using the Shapiro–Wilk test. Differences between groups were analyzed using the Student’s t test or Mann–Whitney U test as appropriate. Categorical variables are reported as frequencies and percentages. Differences between groups were analyzed using the chi-square test or Fisher’s exact test. Variables with *p* < 0.1, as computed in univariate analyses, and other clinical meaningful variables were entered into forward stepwise multivariate logistic regression analyses to identify the association between delirium and poor outcomes (e.g., NIV failure, ICU mortality, and hospital mortality). Bootstrap (1000 samples) was used to validate the association between delirium and poor outcomes. In addition, Cox regression analyses were also performed to retest the association between delirium and poor outcomes. Because delirium can occur at any time during NIV intervention, it was considered to be a time-varying covariate. The time was limited to 65 days as the longest duration of NIV was 65 day. The probability that patients would remain on NIV and in the ICU was analyzed using Kaplan–Meier curves (log-rank test). The threshold for statistical significance was set to *p* < 0.05.

## Results

We enrolled 1083 patients in the study. Of these, 196 (18.1%) developed delirium during NIV intervention. There were no differences between patients with and without delirium in reason for NIV, underlying disease, sex, heart rate, respiratory rate, PaCO_2_, and PaO_2_/FiO_2_ (Table 1). However, delirious patients were older than non-delirious ones, and they also had higher APACHE II score, lower Glasgow coma scale (GCS) score, lower mean arterial pressure, and lower pH. Furthermore, they had higher NIV failure rates, higher ICU mortality, and higher hospital mortality. They also spent longer time on NIV and stayed longer in the ICU and in the hospital (Additional file 3: Fig. S1).

**Table 1:** Patients with and without delirium

Variables	No delirium N = 887	Delirium N = 196	*p*
Age, years	69 (61–77)	78 (71–84)	< 0.01
Female/male	261/626	49/147	0.22
APACHE II score	14 (12–17)	17 (14–20)	< 0.01
Reasons for NIV			
COPD exacerbation	477 (53.8%)	107 (54.6%)	0.67
Pneumonia	184 (20.7%)	48 (24.5%)	
ARDS	63 (7.1%)	11 (5.6%)	
Pulmonary cancer	49 (5.5%)	11 (5.6%)	
Interstitial lung disease	22 (2.5%)	6 (3.1%)	
Asthma	14 (1.6%)	1 (0.5%)	
Heart failure	10 (1.1%)	2 (1.0%)	
OHS	11 (1.2%)	2 (1.0%)	
Pulmonary embolism	15 (1.7%)	0 (0%)	
Others	42 (4.7%)	8 (4.1%)	
Underlying disease			
Hypertension	335 (37.8%)	86 (43.9%)	0.12
Diabetes mellitus	180 (20.3%)	51 (26.0%)	0.08
Chronic heart disease	163 (18.4%)	47 (24.0%)	0.09
Chronic liver disease	36 (4.1%)	4 (2.0%)	0.21
Chronic kidney disease	30 (3.4%)	11 (5.6%)	0.15
Variables collected before NIV			
GCS	15 (15–15)	15 (14–15)	< 0.01
HR, beats/min	111 ± 23	112 ± 22	0.92
RR, breaths/min	30 (26–35)	30 (26–35)	0.75
MAP, mmHg	98 (87–111)	92 (83–106)	< 0.01
pH	7.37 (7.29–7.45)	7.34 (7.26–7.44)	< 0.01
PaCO_2_, mmHg	57 (35–77)	58 (37–81)	0.20
PaO_2_/FiO_2_, mmHg	167 (127–224)	168 (126–243)	0.71
Outcomes			
NIV failure	186 (21.0%)	74 (37.8%)	< 0.01
NIV duration, days	3.7 (1.7–5.9)	6.3 (2.7–11.0)	< 0.01
ICU LOS, days	6.0 (3.8–9.2)	9.0 (4.8–15.2)	< 0.01
Hospital LOS, days	11.0 (6.8–17.0)	14.5 (7.1–21.1)	< 0.01
ICU mortality	127 (14.3%)	65 (33.2%)	< 0.01
Hospital mortality	151 (17.0%)	73 (37.2%)	< 0.01

*NIV* noninvasive ventilation,* COPD* chronic obstructive pulmonary disease,* ARDS* acute respiratory disease syndrome,* OHS* obesity hypoventilation syndrome,* GCS* Glasgow coma scale,* HR* heart rate,* RR* respiratory rate,* MAP* mean arterial pressure,* LOS* length of stay

Delirium was independently associated with NIV failure (odds ratio [OR] = 1.97, 95% confidence interval [CI]: 1.34–2.88), ICU mortality (OR = 2.58, 95%CI: 1.74–3.84), and hospital mortality (OR = 2.55, 95%CI: 1.74–3.75) (Table 2). Bootstrap shows that the OR was 1.97 (95%CI: 1.34–2.97) for NIV failure, 2.58 (1.64–4.08) for ICU mortality, and 2.55 (1.71–3.93) for hospital mortality. These results were also confirmed in patients with and without COPD. Cox regression analyses also showed that delirium was independently associated with NIV failure (Additional files 1, 2: Table S1, S2).

**Table 2:** Results of multivariate analyses of risk factors for NIV failure, ICU mortality, and hospital mortality

Variables	Overall cohort		COPD cohort		Non-COPD cohort	
	OR (95%CI)	*p*	OR (95%CI)	*p*	OR (95%CI)	*p*
Risk factors for NIV failure						
Delirium	1.97 (1.34–2.88)	< 0.01	2.33 (1.27–4.30)	< 0.01	1.84 (1.13–2.99)	0.02
APACHE II score	1.08 (1.03–1.12)	< 0.01	1.09 (1.02–1.17)	0.02	1.10 (1.05–1.15)	< 0.01
GCS	0.78 (0.66–0.91)	< 0.01	–	–	–	–
RR, breaths/min	1.03 (1.00–1.05)	0.02	–	–	–	–
PaCO_2_, mmHg	0.975 (0.968–0.982)	< 0.01	–	–	–	–
PaO_2_/FiO_2_, mmHg	0.996 (0.993–0.998)	< 0.01	–	–	0.995 (0.992–0.998)	< 0.01
Age, years	–	–	1.04 (1.01–1.08)	< 0.01	–	–
HR, beats/min	–	–	1.01 (1.00–1.03)	0.04	–	–
Diabetes mellitus	–	–	–	–	0.57 (0.36–0.91)	0.02
Risk factors for ICU mortality						
Delirium	2.58 (1.74–3.84)	< 0.01	3.64 (1.89–7.01)	< 0.01	2.16 (1.31–3.56)	< 0.01
APACHE II score	1.06 (1.01–1.11)	0.01	1.18 (1.09–1.28)	< 0.01	1.06 (1.01–1.11)	0.03
GCS	0.77 (0.65–0.91)	< 0.01	–	–	–	–
RR, breaths/min	1.04 (1.01–1.06)	< 0.01	–	–	1.03 (1.00–1.06)	0.03
PaCO_2_, mmHg	0.978 (0.970–0.985)	< 0.01	–	–	–	–
PaO_2_/FiO_2_	0.998 (0.995–1.000)	0.04	–	–	0.997 (0.994–1.000)	0.04
Age, years	–	–	1.04 (1.01–1.08)	0.03	–	–
Risk factors for hospital mortality						
Delirium	2.55 (1.74–3.75)	< 0.01	3.34 (1.81–6.18)	< 0.01	2.11 (1.29–3.45)	< 0.01
APACHE II score	1.08 (1.03–1.13)	< 0.01	1.09 (1.02–1.17)	0.02	1.09 (1.04–1.14)	< 0.01
HR, beats/min	1.01 (1.00–1.02)	0.05	1.02 (1.00–1.03)	0.01	–	–
RR, breaths/min	1.03 (1.00–1.05)	0.03	–	–	1.03 (1.00–1.06)	0.02
GCS	0.82 (0.67–0.97)	0.02	–	–	–	–
PaCO_2_, mmHg	0.975 (0.968–0.983)	< 0.01	–	–	–	–
PaO_2_/FiO_2_	–	–	–	–	0.997 (0.995–1.000)	0.05
Age, years	–	–	1.06 (1.03–1.10)	< 0.01	–	–

NIV = noninvasive ventilation, OR = odds ratio, CI = confidence internal, GCS = Glasgow coma scale, HR = heart rate, RR = respiratory rate, COPD = chronic obstructive pulmonary disease

Delirium, sex, age, underlying disease, APACHE II score, GCS scores, heart rate, respiratory rate, pH, PaCO_2_, and PaO_2_/FiO_2_ were entered into multivariate analyses to identify independent risk factors for NIV failure, ICU mortality, and hospital mortality

**Fig. 1:**
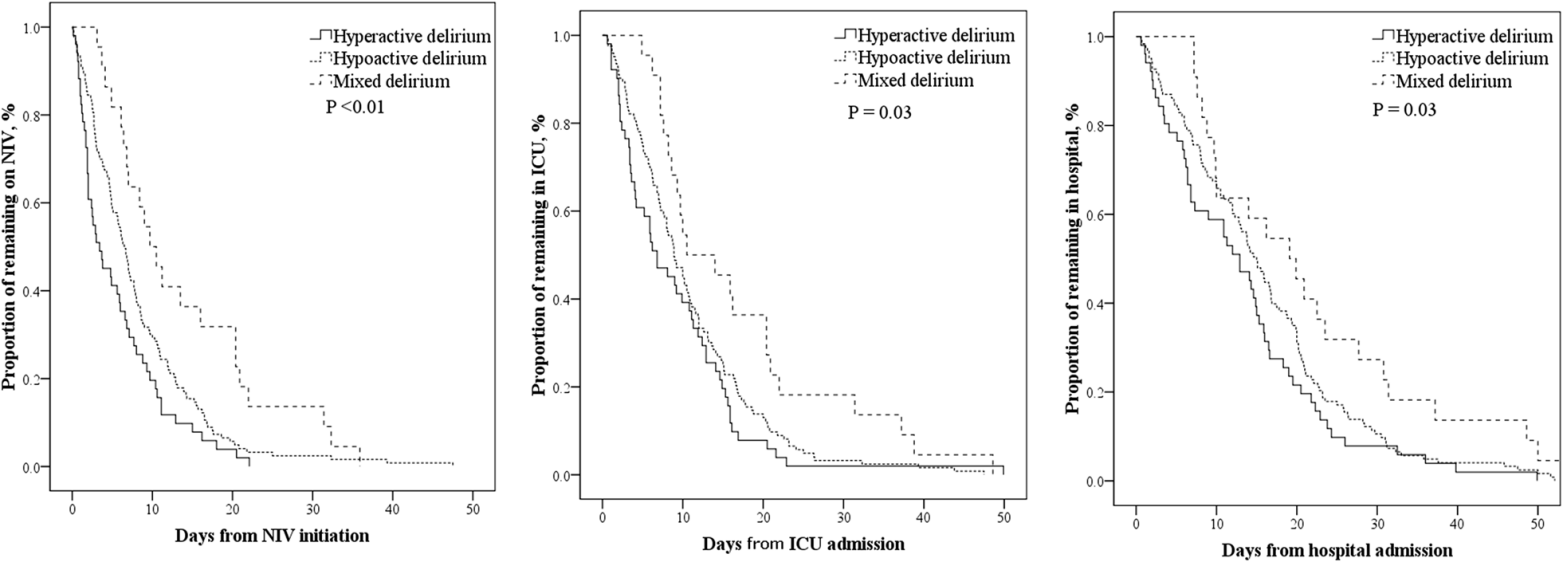
Resource use among patients with hyperactive, hypoactive, and mixed delirium

Among the delirious patients, 51 (26%) developed hyperactive delirium, 123 (63%) developed hypoactive delirium, and 22 (11%) developed mixed delirium (Table 3). There were no differences in NIV failure, ICU mortality, or hospital mortality among the three groups. Patients with hyperactive delirium had the lowest NIV duration, ICU stay, and hospital stay. However, all three were higher in patients with hypoactive delirium and much higher in those with mixed delirium (Table 3 and Fig. 1).

**Table 3:** Results of subgroup analyses in patients with different subtypes of delirium

Variables	Hyperactive delirium N = 51	Hypoactive delirium N = 123	Mixed delirium N = 22	P
NIV failure	19 (37.3%)	47 (38.2%)	8 (36.4%)	0.98
ICU mortality	16 (31.4%)	41 (33.3%)	8 (36.4%)	0.92
Hospital mortality	17 (33.3%)	47 (38.2%)	9 (40.9%)	0.78
NIV duration, days	3.4 (1.7–8.8)	6.5 (2.9–11.0)	10.1 (6.3–20.5)	< 0.01
ICU LOS, days	6.8 (3.3–14.1)	8.9 (5.0–15.1)	12.3 (8.1–21.2)	< 0.01
Hospital LOS, days	12.9 (5.8–19.0)	15.0 (7.9–21.0)	19.5 (9.5–31.0)	0.03
Delirium days	1 (1–2)	1 (1–3)	7 (3–12)	< 0.01

NIV = noninvasive ventilation, LOS = length of stay

**Fig. 2:**
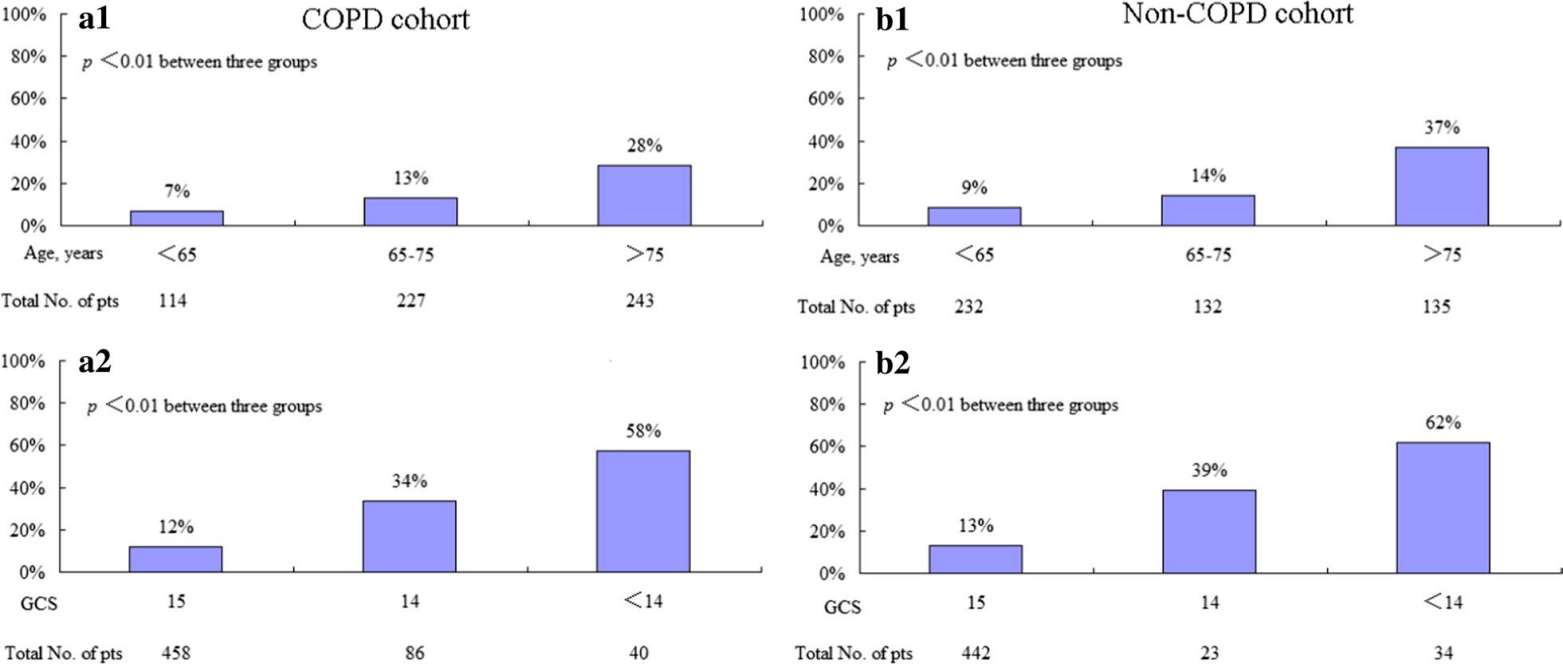
Distributions of delirium in patients at different ages and GCSs. A1 and A2 indicate the COPD cohort. B1 and B2 indicate the non-COPD cohort

Age (OR = 1.06, 95%CI: 1.04–1.08) and APACHE II score (OR = 1.07, 95%CI: 1.03–1.13) were risk factors for delirium, and GCS score (OR = 0.67, 95%CI: 0.56–0.79) and mean arterial pressure (OR = 0.99, 95%CI: 0.98–1.00) were protective factors (Table 4). However, in patients without COPD, only age and GCS were associated with delirium. The distribution of delirium by age and GCS score is presented in Fig. 2. Patients older than 75 years and with a GCS score less than 14 had the highest incidence of delirium. And patients with MAP less than 85 mmHg had higher proportion of delirium than those with MAP more than 85 mmHg (Additional file 4: Fig. S2).

**Table 4:** Results of multivariate analyses of risk factors for delirium

Variables	Overall cohort		COPD cohort		Non-COPD cohort	
	OR (95%CI)	*p*	OR (95%CI)	*p*	OR (95%CI)	*p*
Age, years	1.06 (1.04–1.08)	< 0.01	1.06 (1.03–1.09)	< 0.01	1.07 (1.05–1.09)	< 0.01
GCS	0.67 (0.56–0.79)	< 0.01	0.74 (0.60–0.92)	< 0.01	0.48 (0.37–0.63)	< 0.01
APACHE II score	1.07 (1.03–1.13)	< 0.01	1.08 (1.00–1.15)	0.04	–	–
MAP	0.99 (0.98–1.00)	0.01	0.98 (0.97–0.99)	< 0.01	–	–

NIV = noninvasive ventilation, OR = odds ratio, CI = confidence internal, GCS = Glasgow coma scale, RR = respiratory rate, MAP = mean arterial pressure, COPD = chronic obstructive pulmonary disease

Reasons for NIV, sex, age, underlying disease, APACHE II score, GCS, heart rate, respiratory rate, pH, PaCO_2_, and PaO_2_/FiO_2_ were entered into multivariate analyses to identify independent risk factors for delirium

## Discussion

To the best of our knowledge, this is the largest study of the incidence, characteristics, and outcomes of delirium in NIV patients. A systematic review and meta-analysis investigated 239 NIV patients for incidence of delirium [21]. Its data were extracted from three studies published before 2012 [22–24]. One of those only enrolled COPD patients > 65 years old and did not report how delirium was assessed [22]. In another, delirium was considered if moderate or pronounced encephalopathy was diagnosed [23]. Only one of those three included an assessment of delirium using CAM-ICU (a recommended tool for diagnosis of delirium); however, it only enrolled hypercapnic patients with a median age of 82 years [24]. For these reasons, the results of that systematic review [22] are inaccurate. Another study was published in 2016 [25], which enrolled 153 hypercapnic patients but only reported an association between delirium and 1-year mortality. Due to the small sample size, the relationship among the subtypes of delirium was not reported [21–25]. In our study, we enrolled 1083 NIV patients with different ages and diagnoses. The large sample size enabled us to perform stratification analyses based on diagnosis and age and further explore the relationships among subtypes of delirium.

Hypoactive delirium was the most common subtype reported by Krewulak et al. [2]. In another study, hyperactive delirium was the most common subtype [26]. In our study, hypoactive delirium was the most common subtype, followed by hyperactive delirium and then mixed delirium. In addition, patients with mixed delirium had the largest number of delirium days and the longest ICU stays. To the best of our knowledge, this is the first study to report a distribution among delirium subtypes in NIV patients. Thus, it adds something new to the field that can help clinical practitioners manage NIV patients. For instance, it can reinforce that the incidence of delirium in NIV patients is not low, and delirium is associated with poor outcomes. Awareness of this may lead staff to pay closer more attention to the diagnosis, prevention, and treatment of delirium in NIV patients.

Age, level of consciousness, and disease severity are recognized risk factors for delirium [27–29]. Our study supports this. However, unlike previous studies, we found that mean arterial pressure was a protective factor for delirium. Patients with lower mean arterial pressure were more likely to experience delirium during NIV intervention. Low mean arterial pressure may decrease cerebral blood flow perfusion and lead to neuropsychic symptoms and thus cause delirium. However, this is only a hypothesis, and further exploration is required.

Many studies have found that disease severity, low level of consciousness, increased respiratory rate, and decreased oxygenation are associated with NIV failure [30, 31]. Our study also confirms this. Unlike previous studies, we found that delirium is independently associated with NIV failure both in patients with and without COPD. This provides clinical practitioners with novel insight for understanding NIV failure.

Delirium is highly associated with ICU and hospital mortality [8, 10–12]. Our study supports this in an NIV population. Because delirium patients often have a poor prognosis, it is necessary to prevent delirium. Pain relief, early mobilization, improvement of sleep quality, and minimal noise are promising methods for reducing delirium in NIV patients [32]. Based on the results found in this study, keeping the MAP more than 85 mmHg is a promising strategy to reduce delirium. Further study is encouraged to confirm this issue.

Our study had several limitations. First, it was a single-center observational study performed in an ICU. Because we only enrolled medical patients, the incidence of delirium may have been skewed. Second, we only enrolled patients in whom NIV was used as a first-line intervention. Those who received NIV followed by high-flow nasal cannula or invasive mechanical ventilation were excluded. This means that our results cannot be extrapolated to the total NIV population. Third, we only assessed delirium every morning from NIV initiation to termination. It may be that some patients who were delirious before the start of NIV crossed over in to the non-delirious group if they demonstrated no symptoms of delirium during NIV. Therefore, the incidence of delirium may have been underestimated. Fourth, it was impossible to obtain daily sequential organ failure assessment (SOFA) scores because some of the variables used to calculate SOFA scores were unavailable. Because daily SOFA is superior to APACHE II in terms of disease severity, our use of APACHE II may have reduced the accuracy of our measurements of disease severity.

## Conclusion

The incidence of delirium in the NIV population is high. Hypoactive delirium is the most common subtype in this population. Delirium is associated with increased likelihood of poor outcomes (NIV failure, ICU mortality, and hospital mortality) and use of medical resources (duration of NIV, LOS in the ICU, and LOS in the hospital).

## Supplementary Information

**Additional file 1.** Supplementary Table 1. Results of Cox regression analyses for NIV failure.

**Additional file 2.** Supplementary Table 2. Results of Cox regression analyses for NIV failure, ICU mortality, and hospital mortality among patients with pneumonia/ARDS.

**Additional file 3.** Supplementary Figure 1. Resource use among patients with and without delirium.

**Additional file 4.** Supplementary Figure 2. Distribution of delirium in patients with MAP more than and less than 85 mmHg.

## Data Availability

The datasets analyzed during the current study available from the corresponding author on reasonable request.

## Abbreviations

NIV: Noninvasive ventilation
ICU: Intensive care unit
COPD: Chronic obstructive pulmonary disease
ARDS: Acute respiratory distress syndrome
OHS: Obesity hypoventilation syndrome
GCS: Glasgow coma scale
HR: Heart rate
RR: Respiratory rate
MAP: Mean arterial pressure
LOS: Length of stay
OR: Odds ratio
CI: Confidence interval
CAM-ICU: Confusion Assessment Method for the ICU
RASS: Richmond Agitation-Sedation Scale

## References

[1] Barr J, Fraser GL, Puntillo K (2013). Clinical practice guidelines for the management of pain, agitation, and delirium in adult patients in the intensive care unit. Crit Care Med.

[2] Krewulak KD, Stelfox HT, Leigh JP (2018). Incidence and prevalence of delirium subtypes in an adult ICU: a systematic review and meta-analysis. Crit Care Med.

[3] Hayhurst CJ, Pandharipande PP, Hughes CG (2016). Intensive care unit delirium: a review of diagnosis, prevention, and treatment. Anesthesiology.

[4] Pandharipande PP, Ely EW, Arora RC (2017). The intensive care delirium research agenda: a multinational, interprofessional perspective. Intensive Care Med.

[5] Knauert MP, Gilmore EJ, Murphy TE (2018). Association between death and loss of stage N2 sleep features among critically Ill patients with delirium. J Crit Care.

[6] Devlin JW, Skrobik Y, Gelinas C (2018). Clinical practice guidelines for the prevention and management of pain, agitation/sedation, delirium, immobility, and sleep disruption in adult patients in the ICU. Crit Care Med.

[7] Grahl JJ, Stollings JL, Rakhit S (2018). Antimicrobial exposure and the risk of delirium in critically ill patients. Crit Care.

[8] Salluh JI, Wang H, Schneider EB, et al. Outcome of delirium in critically ill patients: systematic review and meta-analysis. BMJ 2015; 350:h2538. 10.1136/bmj.h2538 PMC4454920 26041151

[9] Ely EW, Inouye SK, Bernard GR (2001). Delirium in mechanically ventilated patients: validity and reliability of the confusion assessment method for the intensive care unit (CAM-ICU). JAMA.

[10] Kim S, Kim JJ, Oh J (2018). Delirium characteristics and outcomes in medical and surgical lnpatients: a subgroup analysis. J Crit Care.

[11] Shehabi Y, Riker RR, Bokesch PM (2010). Delirium duration and mortality in lightly sedated, mechanically ventilated intensive care patients. Crit Care Med.

[12] Milbrandt EB, Deppen S, Harrison PL (2004). Costs associated with delirium in mechanically ventilated patients. Crit Care Med.

[13] Su X, Meng ZT, Wu XH (2016). Dexmedetomidine for prevention of delirium in elderly patients after non-cardiac surgery: a randomised, double-blind, placebo-controlled trial. Lancet.

[14] van den Boogaard M, Slooter AJC, Bruggemann RJM (2018). Effect of haloperidol on survival among critically Ill adults with a high risk of delirium: the REDUCE Randomized Clinical Trial. JAMA.

[15] Skrobik Y, Duprey MS, Hill NS (2018). Low-dose nocturnal dexmedetomidine prevents ICU delirium. A randomized, placebo-controlled trial. Am J Respir Crit Care Med.

[16] Fan L, Zhao Q, Liu Y (2014). Semiquantitative cough strength score and associated outcomes in noninvasive positive pressure ventilation patients with acute exacerbation of chronic obstructive pulmonary disease. Respir Med.

[17] Duan J, Han X, Bai L (2017). Assessment of heart rate, acidosis, consciousness, oxygenation, and respiratory rate to predict noninvasive ventilation failure in hypoxemic patients. Intensive Care Med.

[18] Duan J, Tang X, Huang S (2012). Protocol-directed versus physician-directed weaning from noninvasive ventilation: the impact in chronic obstructive pulmonary disease patients. J Trauma Acute Care Surg.

[19] Sessler CN, Gosnell MS, Grap MJ (2002). The Richmond Agitation-Sedation Scale: validity and reliability in adult intensive care unit patients. Am J Respir Crit Care Med.

[20] Robinson TN, Raeburn CD, Tran ZV (2011). Motor subtypes of postoperative delirium in older adults. Arch Surg.

[21] Charlesworth M, Elliott MW, Holmes JD (2012). Noninvasive positive pressure ventilation for acute respiratory failure in delirious patients: understudied, underreported, or underappreciated? A systematic review and meta-analysis. Lung.

[22] Rozzini R, Sabatini T, Trabucchi M (2006). Non-invasive ventilation for respiratory failure in elderly patients. Age Ageing.

[23] Carlucci A, Richard JC, Wysocki M (2001). Noninvasive versus conventional mechanical ventilation. An epidemiologic survey. Am J Respir Crit Care Med.

[24] Roche Campo F, Drouot X, Thille AW (2010). Poor sleep quality is associated with late noninvasive ventilation failure in patients with acute hypercapnic respiratory failure. Crit Care Med.

[25] Chan KY, Cheng LS, Mak IW (2017). Delirium is a strong predictor of mortality in patients receiving non-invasive positive pressure ventilation. Lung.

[26] Gual N, Inzitari M, Carrizo G (2018). Delirium subtypes and associated characteristics in older patients with exacerbation of chronic conditions. Am J Geriatr Psychiatry.

[27] Janssen TL, Hosseinzoi E, Vos DI (2019). The importance of increased awareness for delirium in elderly patients with rib fractures after blunt chest wall trauma: a retrospective cohort study on risk factors and outcomes. BMC Emerg Med.

[28] Gual N, Morandi A, Perez LM (2018). Risk factors and outcomes of delirium in older patients admitted to postacute care with and without dementia. Dement Geriatr Cogn Disord.

[29] Chaiwat O, Chanidnuan M, Pancharoen W (2019). Postoperative delirium in critically ill surgical patients: incidence, risk factors, and predictive scores. BMC Anesthesiol.

[30] Ozyilmaz E, Ugurlu AO, Nava S (2014). Timing of noninvasive ventilation failure: causes, risk factors, and potential remedies. BMC Pulm Med.

[31] Nava S, Hill N (2009). Non-invasive ventilation in acute respiratory failure. Lancet.

[32] Tang B, Wang XT, Chen WJ (2019). Experts consensus on the management of delirium in critically ill patients. Zhonghua Nei Ke Za Zhi.

