# Supplementary material for: Incidence, characteristics, and outcomes of delirium in patients with noninvasive ventilation: a prospective observational study
Source: BMC Pulm Med. 2021 May 11;21:157. doi: 10.1186/s12890-021-01517-3 (PMC8111378; doi:10.1186/s12890-021-01517-3)
Supplement: Supplementary file 1 — Additional file 1. Supplementary Table 1. Results of Cox regression analyses for NIV failure. [file 12890_2021_1517_MOESM1_ESM.doc]

Supplementary Table 1. Results of Cox regression analyses for NIV failure.

| Variables | Overall cohort |  | COPD cohort |  | Non-COPD cohort |  |
| --- | --- | --- | --- | --- | --- | --- |
|  | HR (95%CI) | *p* | HR (95%CI) | *p* | HR (95%CI) | *p* |
| Delirium | 1.57 (1.34-1.83) | ＜0.01 | 1.76 (1.32-2.34) | ＜0.01 | 1.50 (1.24-1.81) | ＜0.01 |
| APACHE II score | 1.06 (1.03-1.10) | ＜0.01 | 1.13 (1.07-1.19) | ＜0.01 | – | – |
| GCS | 0.80 (0.72-0.90) | ＜0.01 | – | – | – | – |
| RR, breaths/min | 1.02 (1.01-1.04) | 0.01 | – | – | – | – |
| PaCO_2_, mmHg | 0.979 (0.973-0.985) | ＜0.01 | – | – | – | – |
| PaO_2_/FiO_2_, mmHg | 0.996 (0.994-0.998) | ＜0.01 | – | – | 0.996 (0.994-0.998) | ＜0.01 |
| Male | – | – | 2.11 (1.04-4.28) | 0.04 | – | – |
| Age, years | – | – | 1.04 (1.01-1.07) | ＜0.01 | – | – |
| Diabetes mellitus | – | – | – | – | 0.61 (0.42-0.87) | ＜0.01 |

HR = hazard ratio, NIV = noninvasive ventilation, OR = odds ratio, CI = confidence internal, GCS = Glasgow coma scale, RR = respiratory rate, COPD = chronic obstructive pulmonary disease

Delirium, sex, age, underlying disease, APACHE II score, GCS, heart rate, respiratory rate, pH, PaCO_2_, and PaO_2_/FiO_2_ were entered into Cox regression analyses to identify independent risk factors for NIV failure.
