# Supplementary material for: Incidence, characteristics, and outcomes of delirium in patients with noninvasive ventilation: a prospective observational study
Source: BMC Pulm Med. 2021 May 11;21:157. doi: 10.1186/s12890-021-01517-3 (PMC8111378; doi:10.1186/s12890-021-01517-3)
Supplement: Supplementary file 2 — Additional file 2. Supplementary Table 2. Results of Cox regression analyses for NIV failure, ICU mortality, and hospital mortality among patients with pneumonia/ARDS. [file 12890_2021_1517_MOESM2_ESM.doc]

Supplementary Table 2. Results of Cox regression analyses for NIV failure, ICU mortality, and hospital mortality among patients with pneumonia/ARDS

| Variables | NIV failure |  | ICU mortality |  | Hospital mortality |  |
| --- | --- | --- | --- | --- | --- | --- |
|  | HR (95%CI) | *p* | HR (95%CI) | *p* | HR (95%CI) | *p* |
| Delirium | 1.41 (1.10-1.81) | ＜0.01 | 1.41 (1.14-1.75) | ＜0.01 | 1.38 (1.16-1.64) | ＜0.01 |
| APACHE II score | 1.06 (1.02-1.11) | ＜0.01 | 1.08 (1.03-1.14) | ＜0.01 | 1.07 (1.01-1.12) | 0.01 |
| GCS | 0.82 (0.69-0.97) | 0.02 | – | – | – | – |
| RR, breaths/min | – | – | – | – | 1.03 (1.00-1.05) | 0.05 |
| Chronic heart disease | – | – | 1.95 (1.21-3.14) | ＜0.01 | 2.03 (1.28-3.21) | ＜0.01 |

NIV = noninvasive ventilation, HR = hazard ratio, CI = confidence internal, GCS = Glasgow coma scale, RR = respiratory rate

Delirium, sex, age, underlying disease, APACHE II score, GCS, heart rate, respiratory rate, pH, PaCO_2_, and PaO_2_/FiO_2_ were entered into Cox regression analyses to identify independent risk factors.
