# Supplementary figures and images for: Incidence, characteristics, and outcomes of delirium in patients with noninvasive ventilation: a prospective observational study
Source: BMC Pulm Med. 2021 May 11;21:157. doi: 10.1186/s12890-021-01517-3 (PMC8111378; doi:10.1186/s12890-021-01517-3)

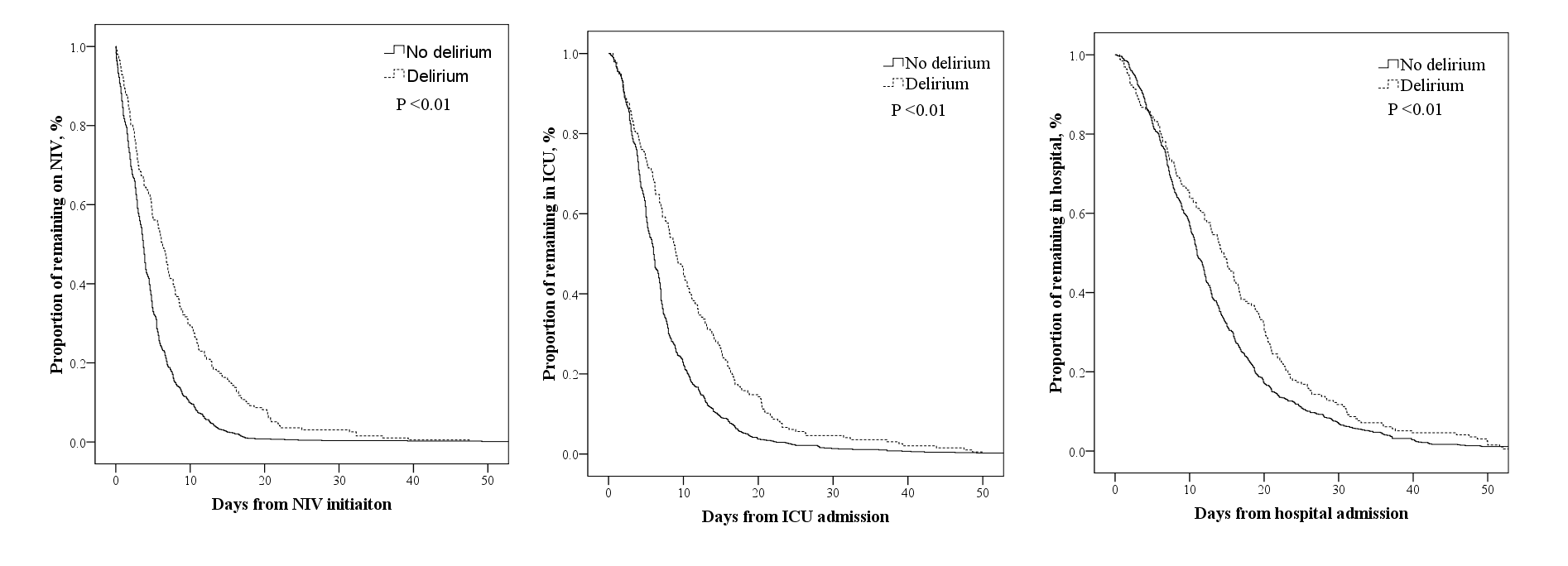

Supplement: Supplementary file 3 — Additional file 3. Supplementary Figure 1. Resource use among patients with and without delirium. [file 12890_2021_1517_MOESM3_ESM.tif]

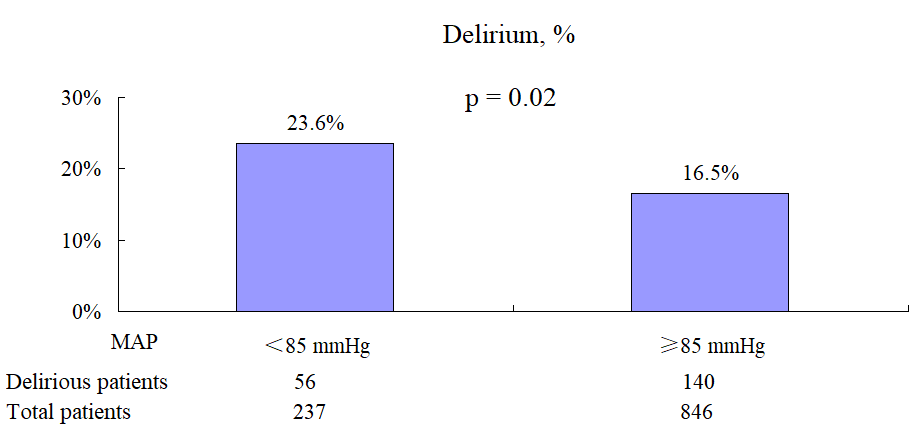

Supplement: Supplementary file 4 — Additional file 4. Supplementary Figure 2. Distribution of delirium in patients with MAP more than and less than 85 mmHg. [file 12890_2021_1517_MOESM4_ESM.tif]
